# 539. Impact of Corticosteroids when Combined with Tocilizumab or Remdesivir for the Treatment of Severe SARS-CoV-2

**DOI:** 10.1093/ofid/ofab466.738

**Published:** 2021-12-04

**Authors:** Michael Rosati, Nikunj M Vyas, Cindy Hou

**Affiliations:** Jefferson Health - New Jersey, Haddon Heights, New Jersey

## Abstract

**Background:**

Tocilizumab (TCZ) and remdesivir (RDV) have both shown benefit for patients with SARS-CoV-2. However, there have been no head to head studies comparing the efficacy of the two therapies. The purpose of this study is to compare clinical outcomes of patients who have received corticosteroids (CS) along with TCZ or RDV.

**Methods:**

This is an IRB approved retrospective observational study completed in a three hospital health system in New Jersey. Patients were included if age was ≥ 18, admitted with SARS-CoV2 infection requiring oxygen. Patients were stratified into two treatment arms; CS + TCZ and CS + RDV. The primary objective was to compare all-cause inpatient mortality (ACIM) based on oxygenation status; nasal cannula (NC), high-flow nasal cannula (HFNC), and invasive mechanical intubation (IMV). Secondary objectives was a snapshot analysis with a focus on clinical improvement (CI) defined as improvement in clinical ordinal scale by 2 or more at end of stay. Additional endpoint included progression to IMV after therapy initiation.

**Results:**

There were total of 1053 patients included (123 in the CS+TCZ arm, 930 in the CS+RDV arm). Oxygen requirements were as follows: In the CS+TCZ arm (NC n=57, HFNC n=26, IMV n=40), and the RD+CS arm (NC n=669, HFN n=159, and IMV n=102). Results from the primary endpoints can be found in Table 1. No statistically significant differences were observed between the two treatment arms. For the secondary objective there were 214 patients included (70 in the CS+TCZ arm and 105 in the CS+RDV arm). For patients receiving NC, no difference seen in CI between two treatment arms (81.4% CS+RDV vs. 81.5% CS+TCZ). In HFNC group more patients in the CS+TCZ group observed CI compared to CS+RDV (68.8% vs. 40%). Less patients requiring HFNC progressed to IMV in CS+TCZ group (25%) compared to CS+RDV (40%).

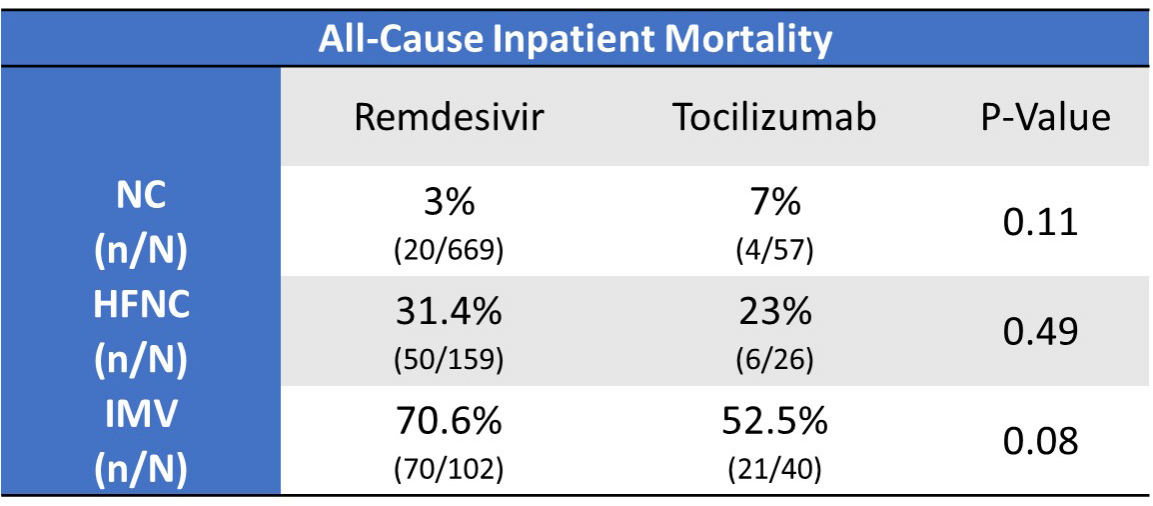

**Conclusion:**

No statistical difference in ACIM was detected between the two treatment arms regardless of baseline oxygenation requirements. There was a trend towards lower ACIM for IMV patients in the CS+TCZ arm compared to the CS+RDV arm. More patients experienced CI in CS+TCZ group compared to CS+RDV in HFNC group. Less HFNC patients also required new IMV in the CS+TCZ arm. Larger studies need to be performed to evaluate a true statistical difference between the two treatment arms.

**Disclosures:**

**All Authors**: No reported disclosures

